# Effects of Enzyme- and Ultrasound-Assisted Treatments on the Recovery of Insoluble-Bound Phenolic Antioxidants from Common Bean Flours

**DOI:** 10.3390/plants15121823

**Published:** 2026-06-12

**Authors:** María José Rojas-Vidal, Miguel A. Varas Condori, María Fernanda Arias-Santé, Samantha Rhein, Raquel Bridi, Miguel Angel Rincón-Cervera, Lee A. Meisel, Nélida Nina, Guillermo Schmeda-Hirschmann, Juana Frias, Adriano Costa de Camargo

**Affiliations:** 1 Institute of Nutrition and Food Technology, University of Chile, Santiago 7830490, Chile; mjrojas11@uc.cl (M.J.R.-V.); mvaras@inta.uchile.cl (M.A.V.C.); ma.fernanda.arias@inta.uchile.cl (M.F.A.-S.); marincer@inta.uchile.cl (M.A.R.-C.); lmeisel@inta.uchile.cl (L.A.M.); 2 Centro de Estudios en Alimentos Procesados (CEAP), Talca 3460000, Chile; samantha.rhein@uoh.cl; 3 Institute of Health Sciences, O’Higgins University, Rancagua 2820000, Chile; 4 Departamento de Química Farmacológica y Toxicológica, Facultad de Ciencias Químicas y Farmacéuticas, Universidad de Chile, Santiago 8380494, Chile; raquelbridi@ciq.uchile.cl; 5 Department of Agronomy, Food Technology Division, University of Almería, 04120 Almería, Spain; 6 Laboratorio de Química de Productos Naturales, Instituto de Química de Recursos Naturales, Campus Lircay, Universidad de Talca, Talca 3460000, Chile; nvnina@umsa.bo (N.N.); schmeda@utalca.cl (G.S.-H.); 7 Área de Biotecnología Microbiana, Instituto de Investigaciones Fármaco Bioquímicas, Facultad de Ciencias Farmacéuticas y Bioquímicas, Universidad Mayor de San Andrés, La Paz 10077, Bolivia; 8 Institute of Food Science, Technology and Nutrition, 28040 Madrid, Spain; frias@ictan.csic.es; 9 Instituto de Ciencias Aplicadas, Universidad Autónoma de Chile, Santiago 7500910, Chile

**Keywords:** common bean, insoluble-bound phenolics, enzyme-assisted extraction, ultrasound-assisted extraction, antioxidant activity, alternative extraction

## Abstract

Common beans contain insoluble-bound phenolic compounds with potential bioactive properties; however, their recovery generally depends on harsh hydrolytic conditions and organic solvents. This study evaluated alternative extraction strategies for the recovery of insoluble-bound phenolic compounds from raw and cooked common bean flours of two Andean varieties (Peumo and Magnum), using Viscozyme L^®^, ultrasound, and pretreatment with ultrasound followed by Viscozyme L^®^. The resulting extracts were characterized in terms of phenolic profile by UPLC-ESI-MS/MS, total phenolic content (TPC), and antioxidant activity. Enzymatic treatment improved the recovery of insoluble-bound phenolic compounds and antioxidant activity compared with the control, while ultrasound alone showed limited effectiveness under the evaluated conditions. The combination of ultrasound pretreatment and Viscozyme L^®^ generally improved recovery of some phenolic compounds and antioxidant-related endpoints relative to control conditions. Cooking generally reduced TPC and antioxidant activity, although the effect on individual phenolic compounds depended on the extraction treatment. Overall, enzyme-assisted extraction, especially when combined with ultrasonic pretreatment, represents a promising strategy for improving the recovery of insoluble phenolic compounds from common bean flour. Further optimization is still needed to improve the sustainability of the process and its industrial applicability.

## 1. Introduction

The common bean (*Phaseolus vulgaris* L.) is a valuable source of protein, essential amino acids, complex carbohydrates, dietary fiber, micronutrients, and bioactive compounds, particularly phenolic compounds, which have been associated with multiple biological activities [1,2]. These compounds are present in both soluble and insoluble forms [3], the latter being those associated with the plant cell wall through physical and chemical interactions [4]. Due to their structural association within the food matrix, insoluble-bound phenolic compounds (IBPs) are less accessible and require specific extraction strategies to be effectively released. IBPs are particularly relevant because they remain associated with structural components of the food matrix, reducing their release during upper gastrointestinal digestion and allowing a substantial proportion to reach the colon, where they may be released during microbial fermentation [4]. Their contribution to antioxidant capacity and their potential biological relevance has increased interest in developing strategies to improve their recovery from plant food matrices. Recent evidence further highlights that the antioxidant potential associated with insoluble-bound phenolics depends strongly on the source material and extraction accessibility, reinforcing the need for optimized recovery approaches [5].

Conventional extraction methods for IBPs typically rely on alkaline or acid hydrolysis, often combined with organic solvents such as diethyl ether or ethyl acetate [3]. While these methods are effective, their use involves harsh conditions and solvents unsuitable for food applications, limiting their applicability in food systems and raising environmental and safety concerns. Consequently, there is growing interest in developing alternative extraction strategies that operate under milder processing conditions and may provide improved compatibility with food applications. Recent studies have emphasized sustainable extraction approaches and improved recovery systems for bound phenolics and bioactive compounds from food matrices and agro-industrial materials [6,7].

Enzyme-assisted extraction has been widely explored as a biotechnological approach to enhance the release of bound compounds from plant matrices [8]. In this context, a well-known enzyme preparation, Viscozyme L^®^, has been recognized as a safe multi-enzyme complex used in the food industry. This complex contains cellulases, arabinases, hemicellulases, β-glucanases, and xylanases, enzymes capable of breaking down cell walls, which, in the case of beans, may enhance the extraction of phenolic compounds from plant tissues [9].

In this regard, a research group that evaluated the effectiveness of Viscozyme L^®^ as a pretreatment for the extraction of phenolic compounds from quinoa observed that this enzyme complex released a greater amount of soluble phenolic compounds than the control group. In addition, phenolic profile characterization showed higher levels for most compounds after Viscozyme L^®^ treatment [10]. Similarly, another research group reported similar results, showing that after Viscozyme L^®^ extraction from the leaves of *Aralia cordata*, sea buckthorn, and industrial hemp, this enzyme complex produced extracts with higher total phenolic content than the control group [11].

In parallel, ultrasound-assisted extraction has been proposed as a physical method that improves mass transfer via cavitation, leading to cell wall disruption and enhanced solvent penetration [12,13,14]. However, the effectiveness of ultrasound-assisted extraction depends strongly on matrix characteristics and processing conditions, including sonication intensity, treatment time, and extraction conditions. Although enzyme-assisted and ultrasound-assisted extraction strategies have been explored in several plant matrices and food systems, including quinoa, soybean, and agro-industrial by-products [10,11,12,13,14], information regarding their application for recovering insoluble-bound phenolic compounds specifically from common bean flours remains limited. Moreover, previous studies in common beans have primarily focused on phenolic characterization following conventional hydrolytic extraction approaches [15], highlighting the need to investigate alternative recovery strategies under milder processing conditions.

In previous work by our research group, the phenolic profile and antioxidant activity of IBPs from common bean flour were characterized in both raw and cooked samples following alkaline hydrolysis, highlighting this fraction as a source of bioactive compounds [15]. However, alkaline hydrolysis requires harsh chemical conditions incompatible with food applications. Therefore, there is a need to explore alternative extraction strategies that allow for the efficient recovery of these compounds under milder processing conditions.

In this context, the present study aimed to evaluate enzyme-assisted and ultrasound-assisted extraction strategies, applied individually and in combination, as alternatives for recovering insoluble-bound phenolic compounds from raw and cooked common bean flours. Special emphasis was placed on determining whether ultrasound pretreatment could improve enzymatic extraction efficiency and enhance the recovery of phenolic compounds and antioxidant activity. Given the limited information available on combined ultrasound and enzymatic strategies for the recovery of insoluble phenolic compounds from common bean flours, this work seeks to provide new evidence on alternative extraction methods for legume matrices. We hypothesized that ultrasound pretreatment would enhance enzyme accessibility to the bean matrix, facilitating the release of insoluble-bound phenolic compounds and improving phenolic recovery and antioxidant activity compared with individual treatments.

## 2. Results

### 2.1. Treatments and Phenolic Profile

The effect of the different treatments on the phenolic profile of bean flours from the Peumo and Magnum varieties is shown in Table 1 and Table 2, respectively. Identification details such as precursor ions, product ions, retention time, and others are presented as Supplementary Materials (Table S1). Regarding the Peumo variety, treatment with Viscozyme L^®^ or the combination of ultrasound pretreatment and Viscozyme L^®^ showed higher concentrations of several phenolic compounds compared to the control and ultrasound treatments.

In particular, the most abundant phenolic acid was ferulic acid for Peumo variety, with higher concentrations after extraction with Viscozyme L^®^ (4.26 and 4.80 μg/g dry sample for raw and cooked beans, respectively), followed by the combination of ultrasound pretreatment and Viscozyme L^®^ (3.89 and 4.01 μg/g), ultrasound alone (0.29 and 0.29 μg/g), and the control (0.38 and 0.25 μg/g), with no significant differences between the latter two groups. Furthermore, for all treatments except the control, no significant differences were observed between raw and cooked samples of this compound. For most phenolic acids, no significant differences were observed between samples subjected to ultrasound and the control group. However, the combination of ultrasound pretreatment and Viscozyme L^®^ showed higher concentrations of certain phenolic acids, including caffeic acid, the second most abundant phenolic acid.

Regarding the effect of cooking on the phenolic acid concentrations for the different treatments, the effects were variable in the Peumo variety. In the case of ultrasound treatment, cooking resulted in decreases in two compounds, increased the concentration of four compounds—among which p-coumaric acid showed the highest increase (190%)—and led to no significant differences in one compound. For treatment with Viscozyme L^®^, no decreases were observed in any phenolic acid, with increases in three compounds, while showing no significant differences in four others. In the case of the combination of ultrasound pretreatment and Viscozyme L^®^, similar variability was observed, with decreases in three compounds, an increase in one, and no significant differences in three others.

Regarding the flavonoids of the Peumo variety, higher concentrations were observed for all treatments compared with phenolic acids, being about seven times higher for the raw variety and three times higher for the cooked variety. The major flavonoid identified was kaempferol, reaching 49.70 and 23.42 μg/g dry sample for raw and cooked samples, respectively, after the combination of ultrasound pretreatment and Viscozyme L^®^. When the two major flavonoids—kaempferol and quercetin—were compared with the control group, they were 12 and 7 times higher, respectively, for raw samples, and 65 and 87 times higher, respectively, for cooked samples.

The behavior of flavonoid content, depending on the type of treatment, followed the order: ultrasound + Viscozyme L^®^ > Viscozyme L^®^ > Control > Ultrasound. Overall, total flavonoid content in the Peumo variety was markedly higher in the ultrasound + Viscozyme L^®^ treatment than in the control, reaching approximately 3.3-fold higher values in raw samples and 8.7-fold higher values in cooked samples. Cooking resulted in significant decreases in all identified flavonoids, with reductions ranging from 33% for taxifolin after treatment with Viscozyme L^®^ to 95% for quercetin in the control group.

Regarding the phenolic profile of the Magnum variety, as in the Peumo variety, the major phenolic acid was ferulic acid, reaching 4.17 and 5.43 μg/g dry sample for raw and cooked samples, respectively, after treatment with Viscozyme L^®^. Another phenolic acid identified was caffeic acid, which reached 3.15 and 2.45 μg/g dry sample for raw and cooked samples, respectively, when treated with ultrasound + Viscozyme L^®^. Regarding the behaviour of phenolic acid content depending on the type of treatment for the Magnum variety, the following order was observed—ultrasound + Viscozyme L^®^ > Viscozyme L^®^ > Ultrasound > Control—although ultrasound and control treatments did not show significant differences between them.

The effect of cooking on phenolic acid concentrations was variable; for the same compound, cooking resulted in an increase, a decrease, or no significant differences, depending on the extraction method. For instance, no significant differences were observed in syringic acid after ultrasound treatment, increased after Viscozyme L^®^ and ultrasound + Viscozyme L^®^ treatments, and decreased in the control group.

As for the identified flavonoids, higher concentrations were observed compared to phenolic acids. Overall, total flavonoid content for all treatments was about six times higher for the raw variety and three times higher for the cooked variety. The behavior of flavonoid concentrations followed a similar pattern to that observed in the Peumo variety: ultrasound + Viscozyme L^®^ > Viscozyme L^®^ > Ultrasound > Control. When comparing the treatment that yielded the highest flavonoid extraction (ultrasound + Viscozyme L^®^) with the control, kaempferol levels were 15 times higher in both raw and cooked samples, while quercetin was 73 and 72 times higher for raw and cooked samples, respectively.

A similar pattern was observed in relation to the effect of cooking on flavonoid content: all flavonoids were significantly affected, with decreases ranging from 12% for myricetin after treatment with Viscozyme L^®^ to 91% for catechin after the combined ultrasound + Viscozyme L^®^ treatment. Catechin showed the greatest decrease, with an average reduction of 83% for Peumo and 86% for Magnum, while the least affected was taxifolin, with reductions of 36% and 32%, respectively, across treatments.

**Figure 1:**
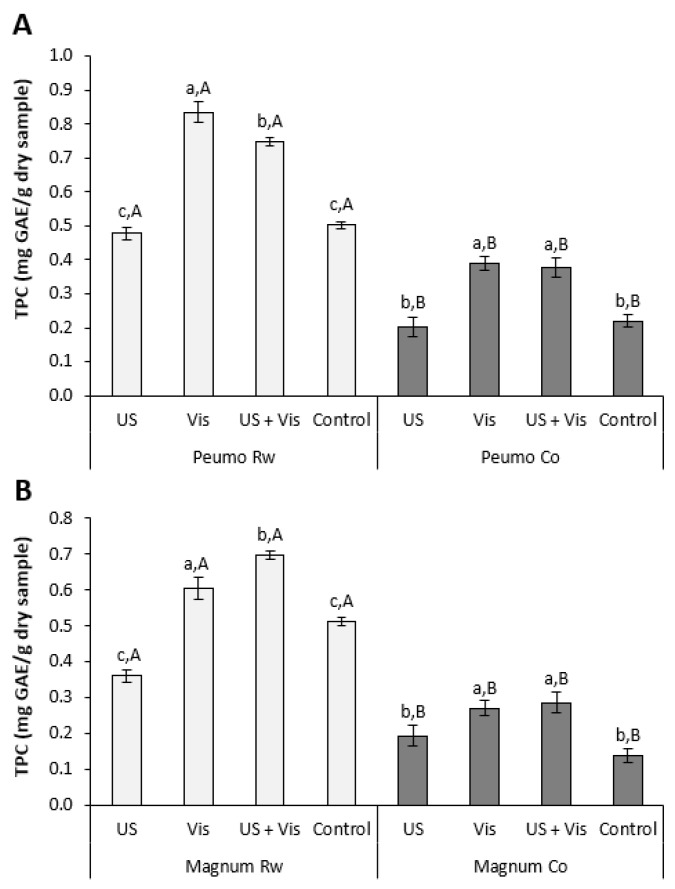
Effect of enzyme treatment, ultrasound, and the combination of ultrasound + enzyme treatment on total phenolic content for each treatment: raw and cooked Peumo variety (**A**); raw and cooked Magnum variety (**B**). Treatments included are: Ultrasonic (US), Viscozyme L^®^ (Vis), Ultrasound pretreatment + Viscozyme L^®^ (US + Vis), Control. TPC: Total phenolic compounds. GAE: Gallic acid equivalents. Data are mean ± standard deviation (n = 9). Different lowercase letters above the bars represent significant differences between treatments for each of the raw and cooked varieties according to one-way ANOVA followed by Tukey’s post hoc test. Different uppercase letters above the bars represent significant differences between raw and cooked samples for each of the treatments according to Student’s *t*-test.

Regarding the total phenolic content (TPC) of both varieties for each treatment (Figure 1), cooking resulted in lower TPC values in both varieties across all treatments. For the Peumo variety, the highest TPC values were observed following Viscozyme L^®^ treatment (0.84 and 0.39 mg GAE/g dry sample for raw and cooked samples, respectively), whereas ultrasound + Viscozyme L^®^ yielded 0.75 and 0.38 mg GAE/g dry sample. For the Magnum variety, a different response pattern was observed. Viscozyme L^®^ treatment resulted in 0.61 and 0.27 mg GAE/g dry sample for raw and cooked samples, respectively, whereas ultrasound + Viscozyme L^®^ treatment resulted in 0.70 and 0.29 mg GAE/g dry sample.

These findings indicate that treatment effectiveness depended on bean variety. While the combined ultrasound pretreatment and Viscozyme L^®^ treatment resulted in the highest TPC values in Magnum, Viscozyme L^®^ alone yielded the highest TPC values in Peumo. Additionally, higher concentrations of flavonoids were observed compared to phenolic acids.

### 2.2. Treatments and Antioxidant Activity

The effect of the different treatments on the antioxidant activity of the two bean varieties is presented in Table 3 and Table 4.

For the Peumo variety, antioxidant activity responses varied according to the assay evaluated. The FRAP assay showed the highest antioxidant activity values, reaching 23.68 and 8.06 μmol TE/g dry sample after ultrasound + Viscozyme L^®^ treatment for raw and cooked beans, respectively, and 17.42 and 12.49 μmol TE/g dry sample after Viscozyme L^®^ treatment. In general, Viscozyme L^®^ treatment and ultrasound + Viscozyme L^®^ pretreatment tended to show higher antioxidant activity values than the control treatment; however, treatment responses differed among assays. Ultrasound treatment alone generally did not show significant differences compared with the control.

The Magnum variety exhibited similar results. The FRAP assay showed values of 24.05 and 6.88 μmol TE/g dry sample after ultrasound + Viscozyme L^®^ treatment, and 24.12 and 8.38 μmol TE/g dry sample after Viscozyme L^®^ treatment for raw and cooked beans, respectively. As observed in the Peumo variety, antioxidant responses depended on both extraction treatment and assay type. Viscozyme L^®^ treatment and ultrasound + Viscozyme L^®^ treatment generally yielded higher antioxidant activity values relative to the control; however, no single treatment consistently showed the highest antioxidant activity across all assays. Ultrasound treatment alone generally did not differ significantly from the control.

Finally, regarding the effect of cooking on antioxidant activity, raw samples of both Peumo and Magnum varieties showed significantly higher antioxidant activity values than their cooked counterparts across all assays. Among the antioxidant assays evaluated, the ORAC assay showed the lowest average reduction after cooking, with decreases of 35.9% and 40.7% in the Peumo and Magnum varieties, respectively.

## 3. Discussion

The extraction of insoluble-bound phenolic compounds (IBPs) from legume matrices presents a technological and analytical challenge due to their strong association with structural components of the cell wall, such as cellulose, hemicellulose, and lignin. These interactions limit their extraction by conventional aqueous or mild-solvent methods. While alkaline and acid hydrolysis remain effective for IBP release, their use involves extreme conditions and organic solvents incompatible with food applications. Therefore, the development of alternative extraction strategies that balance efficiency and sustainability remains a key research priority [7], particularly considering increasing evidence supporting the importance of insoluble-bound phenolics as contributors to antioxidant potential depending on food source characteristics [5].

In this context, the present study evaluated enzymatic and ultrasound-assisted approaches, individually and in combination, to improve the recovery of IBPs from common bean flours. Notably, the results demonstrate that enzymatic treatment with Viscozyme L^®^ significantly improved the extraction of phenolic compounds and antioxidant activity compared to the control. This observation is consistent with previous findings showing that multi-enzyme complexes enhance phenol release by hydrolyzing structural polysaccharides and breaking down the physical barriers that retain bound compounds [10,16]. Enzymatic degradation of cellulose, hemicellulose, and β-glucans increases matrix porosity and facilitates solvent access to esterified and trapped phenolic compounds, a process that is particularly relevant in legumes, where IBPs are predominantly associated with structural polysaccharides of the cell wall (e.g., cellulose, hemicellulose, and lignin) [7].

In line with these findings, the magnitude of the observed increase in antioxidant activity is consistent with studies on lentil hulls and rice bran, in which enzymatic treatments significantly improved both phenolic yield and antioxidant capacity [16,17]. These results reinforce the suitability of enzyme-assisted extraction as a strategy for recovering bioactive compounds from complex plant matrices.

In contrast, ultrasound treatment alone did not consistently improve the extraction of phenolic compounds or antioxidant activity under the evaluated conditions. In most cases, no significant differences were observed between ultrasound-treated samples and the control. This suggests that the effectiveness of ultrasound-assisted extraction depends strongly on processing parameters and matrix characteristics, as previously reported [13,18]. Previous studies have shown that ultrasound effects may vary according to sonication intensity, exposure time, and physicochemical properties of plant materials, which influence cavitation phenomena and mass transfer efficiency. While cavitation has been reported to promote physical disruption of plant matrices and enhance solvent accessibility, these effects may not be sufficient to release phenolic compounds that remain chemically bound to structural components [19].

Furthermore, ultrasound treatment has been associated in previous studies with localized heating phenomena and the formation of reactive species, which under certain operating conditions could contribute to the degradation or transformation of sensitive compounds [13,19]. However, these mechanisms were not directly evaluated in the present study and therefore should be considered only as possible explanations. Consequently, the limited effectiveness of ultrasound observed here should be interpreted within the context of the evaluated matrix and operating conditions rather than as an intrinsic limitation of the technique.

A key finding of this study is the improved performance observed when ultrasound pretreatment was combined with enzymatic extraction. Although ultrasound alone was not effective under the evaluated conditions, its use as a pretreatment improved the subsequent enzymatic release of phenolic compounds, suggesting a complementary effect between both processes. Previous studies have proposed that ultrasound pretreatment may promote physical changes in plant matrices that facilitate enzyme accessibility to substrate-binding sites [13,14]. Subsequent enzymatic hydrolysis may further degrade structural polysaccharides, promoting the release of bound phenolic compounds [16]. However, these mechanisms were not directly evaluated in the present study and therefore should be interpreted as potential explanations supported by the previous literature.

This complementary effect has also been observed in agro-industrial byproducts, such as cashew nut testa and pomelo peel by-products, where combined treatments yielded higher phenolic levels than individual methods [20,21]. The present results are consistent with this proposed complementary effect and highlight the potential value of integrating physical and biochemical processes to improve extraction efficiency.

The effect of cooking on phenolic composition and antioxidant activity was complex and depended on both the compound and the extraction method. While an overall decrease in total phenolic content and antioxidant activity was observed after cooking, some individual phenolic acids showed increases or remained unchanged. These contrasting trends can be explained by the simultaneous occurrence of multiple phenomena, including the thermal degradation of labile compounds, disruption of the cell wall matrix, and the release of bound phenolic compounds [22,23].

Thermal processing can improve the extractability of certain phenolic compounds by weakening their interactions with the matrix, while simultaneously promoting the oxidation or structural transformation of others. This dual effect has been documented in legumes, where cooking can enhance the release of bound phenolic compounds and induce the degradation of heat-labile compounds [22]. These findings underscore the need for compound-specific interpretation, rather than generalizations about phenolic compound losses during cooking.

From a sustainability perspective, enzymatic and ultrasound-assisted extraction approaches have been proposed as alternative strategies to conventional hydrolysis-based methods. However, it is important to critically evaluate the entire extraction workflow. In the present study, while the matrix treatment stages were performed in aqueous media, recovery, isolation, and quantification of phenolic compounds required the use of organic solvents such as ethyl acetate and methanol for analytical purposes. Therefore, the analytical workflow employed here cannot be considered fully aligned with green extraction principles. Similar limitations have been highlighted in recent reviews, emphasizing the importance of developing extraction systems that further reduce solvent requirements and improve the valorization of phenolic-rich food materials through sustainable processing approaches [6,7].

It should be noted that the organic solvents employed in this study were used exclusively for analytical purposes, including isolation and quantification of phenolic compounds, and do not necessarily reflect conditions required for potential food processing applications. The extraction approaches evaluated in this work aim to reduce reliance on harsh hydrolysis conditions traditionally employed for insoluble-bound phenolic recovery.

Overall, this study demonstrates that enzyme-assisted extraction, especially when combined with ultrasound pretreatment, shows potential as a strategy under the evaluated experimental conditions for improving the recovery of insoluble phenolic compounds from common bean flour. The findings contribute to a better understanding of the factors influencing the release of these compounds and support the potential valorization of legumes as sources of bioactive compounds. It should be noted that the extraction conditions evaluated in this study were selected based on previous studies and were not intended to represent optimized processing parameters. Therefore, the present work should be interpreted as a comparative evaluation of the tested extraction strategies under the specific conditions examined. Further optimization of ultrasound and enzymatic extraction parameters, together with the development of more sustainable extraction workflows, will be necessary before assessing potential industrial applications.

## 4. Materials and Methods

### 4.1. Reagents and Solvents

Sodium acetate, gallic acid, ABTS (2,2′-azino-bis(3-ethylbenzothiazoline-6-sulfonic acid)), sodium carbonate, iron chloride (FeCl_3_), AAPH (2,2′-azobis(2-amidinopropane) dihydrochloride), DPPH (2,2-diphenyl-1-picrylhydrazyl radical), fluorescein, Folin–Ciocalteu reagent, potassium persulfate, TPTZ (2,4,6-tri(2-pyridyl)-1,3,5-triazine), Trolox (6-hydroxy-2,5,7,8-tetramethylchroman-2-carboxylic acid), and Viscozyme L^®^ were purchased from Sigma-Aldrich (St. Louis, MO, USA). Ethyl acetate, glacial acetic acid, hydrochloric acid, and methanol (analytical and HPLC grades) were purchased from Merck (Darmstadt, Germany).

### 4.2. Samples

Common bean (*Phaseolus vulgaris* L.) seeds from the Peumo and Magnum varieties were obtained from the Maule Region, central Chile. Bean sourcing and preprocessing procedures, including storage conditions (20 °C, fabric bags), cooking, lyophilization, and flour preparation, were performed by Nina et al. [24] prior to the experiments conducted in the present study. Briefly, whole beans were soaked overnight in water (1:3, *w*/*v*), the soaking water was replaced prior to cooking, and samples were boiled in tap water for 60 min. After cooking, the decoction water was discarded, and cooked beans were frozen, lyophilized, and milled before extraction as previously described [15,24].

### 4.3. Experimental Design

The experimental design included four treatment conditions for each bean variety: (i) control (no ultrasound, no enzyme), (ii) ultrasound alone, (iii) enzyme-assisted extraction, and (iv) ultrasound pretreatment followed by enzymatic extraction. Each condition was evaluated in both raw and cooked samples.

### 4.4. Ultrasound-Assisted Extraction

Ultrasound-assisted extraction was carried out with modifications from previously reported methods [19]. Briefly, 5 g of bean flour were suspended in distilled water (1:20, *w*/*v*) and homogenized under constant stirring. The suspension was subjected to probe sonication (20 kHz; VCX750, Sonics & Materials, Newtown, CT, USA) for 5 min using 20 s pulses with 5 s intervals, at 47% amplitude (50 W).

The ultrasonic processor was set to automatically stop if the temperature reached 40 °C; however, during the experiments, the temperature remained below this threshold, and no active cooling was required.

After sonication, samples were immediately frozen and lyophilized (MFD-1050M). The dried material was stored at −18 °C until further enzymatic treatment or extraction.

The released phenolic compounds were extracted five times with ethyl acetate (1:1, *v*/*v*) at pH 2, as previously described [25]. Between each wash, the sample was centrifuged (Z 206 A, HERMLE, Wehingen, Germany) at 4000× *g* for 5 min, and the supernatant was collected. The solvent was evaporated to dryness under vacuum (HB eco, IKA-Werke, Staufen, Germany), and the extract was reconstituted in 5 mL of HPLC-grade methanol. Samples were stored at −20 °C until analysis.

Ultrasound conditions were selected based on previous studies reporting efficient recovery of phenolic compounds under moderate sonication conditions while minimizing excessive thermal exposure and potential degradation of sensitive compounds [19].

### 4.5. Enzyme-Assisted Extraction

Enzyme-assisted extraction was performed on both untreated (non-sonicated) and ultrasound-pretreated samples to evaluate the individual and combined effects of each treatment.

Enzyme-assisted extraction was performed as described previously, with slight modifications [26]. Briefly, 4 g of sample (untreated or sonicated and lyophilized) were suspended in 40 mL of Viscozyme L^®^ solution (2% *w/v* in 0.1 M acetate buffer, pH 4). The mixture was incubated at 37 °C for 16 h under constant agitation at 150 rpm in a thermostatic bath (LSB-130S, LabTech, Sorisole, Italy).

After incubation, the mixture was acidified to pH 2.0 with 6 M HCl. Released phenolic compounds were extracted five times with ethyl acetate (1:1, *v*/*v*), as previously described [27]. Between each wash, the sample was centrifuged (Z 206 A, HERMLE, Wehingen, Germany) at 4000× *g* for 5 min, and the supernatant was collected. The solvent was evaporated to dryness under vacuum at 40 °C (HB eco, IKA-Werke, Staufen, Germany), and the extract was reconstituted in 5 mL of HPLC-grade methanol. Samples were stored at −20 °C until further analysis.

Control samples consisted of bean suspensions treated under identical conditions using acetate buffer without enzyme.

### 4.6. Total Phenolic Content (TPC)

Total phenolic content was evaluated following a previously described procedure with modifications [28]. Briefly, 150 μL of each sample was evaporated to dryness and reconstituted in distilled water. Then, 450 μL of distilled water and 2 mL of Folin–Ciocalteu reagent were added. After 3 min, 400 μL of 20% sodium carbonate solution was added. Samples were incubated for 30 min at 37 °C in a thermoblock (HB120-S, DLAB, Beijing, China) protected from light. Absorbance was measured at 765 nm using a UV–Vis spectrophotometer (N4S UV–Vis Precise Perfect, Hinotek, Ningbo, China). Gallic acid was used to construct the calibration curve (0–280 mg/L). Results were expressed as milligrams of gallic acid equivalents per gram of dry sample (mg GAE/g dry sample).

### 4.7. Antioxidant Activity

Prior to each antioxidant assay, extracts were appropriately diluted to fall within the linear range of the method. For the ORAC assay, dilutions were performed using phosphate buffer (75 mM, pH 7.4), whereas for FRAP, ABTS, and DPPH assays, methanol was used as the dilution solvent.

#### 4.7.1. Oxygen Radical Absorbance Capacity (ORAC) Assay

The ORAC assay was performed as described previously, with minor modifications [25]. Briefly, 175 μL of phosphate buffer (75 mM, pH 7.4) was mixed with 30 μL of fluorescein and 20 μL of diluted sample. The mixture was incubated for 20 min at 37 °C, followed by the addition of 25 μL of 10 mM AAPH. The reaction was incubated for 2 h at 37 °C. A fluorescence microplate reader (Infinite 200 Pro, TECAN, Männedorf, Switzerland) was used at excitation 480 nm and emission 510 nm. Results were expressed as μmol Trolox equivalents per gram of dry sample (μmol TE/g dry sample).

#### 4.7.2. Ferric Reducing Antioxidant Power (FRAP) Assay

The FRAP assay was conducted as described previously, with minor modifications [25]. Acetate buffer (0.3 M, pH 3.6) was mixed with TPTZ (10 mM) and ferric chloride (20 mM) in a 10:1:1 (*v*/*v*/*v*) ratio. Then, 2700 μL of this solution was combined with 30 μL of the diluted sample and incubated for 30 min at 37 °C in the dark. Absorbance was measured at 593 nm using a UV–Vis spectrophotometer (N4S UV–Vis Precise Perfect, Hinotek, Ningbo, China). Values were expressed as μmol Trolox equivalents per gram of dry sample (μmol TE/g dry sample).

#### 4.7.3. DPPH Radical Scavenging Activity Assay

DPPH radical scavenging activity was measured as described previously, with slight modifications [29]. Briefly, 1950 μL of DPPH solution (0.06 mM) were mixed with 50 μL of the diluted sample and incubated for 45 min protected from light. Absorbance was read at 515 nm using a UV–Vis spectrophotometer (N4S UV–Vis Precise Perfect, Hinotek, Ningbo, China). Results were expressed as μmol Trolox equivalents per gram of dry sample (μmol TE/g dry sample).

#### 4.7.4. ABTS Radical Cation Scavenging Activity Assay

ABTS radical cation scavenging activity was carried out as described previously, with slight modifications [28]. Briefly, ABTS solution (7 mM) was mixed with potassium persulfate (2.45 mM) and incubated in the dark for 14 h to generate the ABTS radical cation. Then, 3 mL of diluted ABTS solution (absorbance 0.70 ± 0.02 at 734 nm) was mixed with 30 μL of diluted sample and incubated for 6 min protected from light. Absorbance was read at 734 nm using a UV–Vis spectrophotometer (N4S UV–Vis Precise Perfect, Hinotek, Ningbo, China). Results were expressed as μmol Trolox equivalents per gram of dry sample (μmol TE/g dry sample).

### 4.8. UPLC–ESI–MS/MS Analysis

Phenolic compounds were identified and quantified as previously described [25] using an ABSciex Triple Quad 4500 mass spectrometer equipped with an electrospray interface (TurboV) and coupled to an Eksigent Ekspert Ultra LC100 system with an Ultra LC100-XL autosampler (AB/Sciex, Concord, ON, Canada). Electrospray ionization was performed in negative mode under the following parameters: curtain gas (CUR) = 30 psi; collision gas (CAD) = 10 psi; ion spray voltage (IS) = −4500 V; temperature (TEM) = 650 °C; ion source gas 1 (GS1) = 50 psi; ion source gas 2 (GS2) = 50 psi; entrance potential (EP) = 10 V.

Chromatographic separation was achieved using gradient elution with (A) 0.1% formic acid and (B) methanol as the mobile phases, following this program: 0–1 min, 5% B; 1–12 min, 5–50% B; 12–13 min, 50% B; 13–14 min, 50–5% B; and 14–15 min, 5% B. The injection volume was 10 μL, with a flow rate of 0.5 mL/min, using a LiChrospher 100 RP-18 column (125 mm × 4 mm i.d., 5 μm; Merck, Darmstadt, Germany) maintained at 40 °C. Calibration curves were constructed using commercially available standards.

Limits of detection (LOD), limits of quantification (LOQ), and correlation coefficients (r^2^) for each standard were as follows: gallic acid (LOD = 41 ppb, LOQ = 124 ppb, r^2^ = 0.9988); caffeic acid (LOD = 142 ppb, LOQ = 430 ppb, r^2^ = 0.9976); p-coumaric acid (LOD = 124 ppb, LOQ = 377 ppb, r^2^ = 0.9911); ferulic acid (LOD = 110 ppb, LOQ = 334 ppb, r^2^ = 0.9944); syringic acid (LOD = 55 ppb, LOQ = 167 ppb, r^2^ = 0.9995); sinapic acid (LOD = 120 ppb, LOQ = 364 ppb, r^2^ = 0.9984); catechin (LOD = 49 ppb, LOQ = 150 ppb, r^2^ = 0.9997); epicatechin (LOD = 83 ppb, LOQ = 252 ppb, r^2^ = 0.9991); luteolin (LOD = 136 ppb, LOQ = 412 ppb, r^2^ = 0.9965); kaempferol (LOD = 390 ppb, LOQ = 1181 ppb, r^2^ = 0.9905); myricetin (LOD = 180 ppb, LOQ = 545 ppb, r^2^ = 0.9956); quercetin (LOD = 67 ppb, LOQ = 203 ppb, r^2^ = 0.9969); and rutin (LOD = 249 ppb, LOQ = 756 ppb, r^2^ = 0.9939).

### 4.9. Statistical Analysis

All experiments were performed using three independent extractions per treatment. Each extract was analyzed in triplicate for all assays. Therefore, the reported values represent the mean of nine measurements (n = 9), expressed as mean ± standard deviation. Independent extractions were considered as biological replicates, while repeated measurements were treated as technical replicates.

Statistical analysis was performed using STATA version 15.0. Normality of the data distribution was assessed using the Shapiro–Wilk test, and homogeneity of variances was evaluated using Levene’s test.

Differences between extraction treatments were analyzed using one-way ANOVA followed by Tukey’s *post hoc* test. Differences between raw and cooked samples were evaluated using Student’s *t*-test. Statistical significance was established at *p* < 0.05.

## 5. Conclusions

Common bean flours are an important source of insoluble-bound phenolic compounds, although their recovery depends strongly on the extraction strategy employed. In this study, Viscozyme L^®^ treatment improved phenolic release and antioxidant activity relative to the control, whereas ultrasound alone showed limited efficacy under the evaluated conditions. Ultrasound pretreatment combined with Viscozyme L^®^ generally enhanced performance across multiple evaluated endpoints, supporting the use of combined physical and enzymatic approaches to improve the recovery of insoluble-bound phenolic compounds from legume matrices. Cooking generally reduced total phenolic content and antioxidant activity, although responses varied among individual compounds. Future studies should focus on process optimization and more sustainable recovery workflows to facilitate potential food and industrial applications.

## Figures and Tables

**Table 1:** Identification and concentration by UPLC-MS/MS (μg/g dry weight) of the insoluble phenolic fraction of common bean (*Phaseolus vulgaris* L.) of the Peumo variety, under different extraction treatments.

	Ultrasound	Viscozyme L^®^	Ultrasound + Viscozyme L^®^	Control
**Phenolic acids**				
	**Gallic acid**			
Raw	0.10 ± 0.00 bC	0.04 ± 0.00 bB	0.05 ± 0.00 A	ND
Cooked	0.12 ± 0.00 aA	0.06 ± 0.00 aB	ND	ND
	**Syringic acid**			
Raw	0.01 ± 0.00 aC	0.03 ± 0.00 bB	0.04 ± 0.00 bA	0.01 ± 0.00 aC
Cooked	0.01 ± 0.00 bC	0.04 ± 0.00 aB	0.06 ± 0.00 aA	0.01 ± 0.00 aC
	**Ferulic acid**			
Raw	0.29 ± 0.00 aC	4.26 ± 0.19 aA	3.89 ± 0.09 aB	0.38 ± 0.01 aC
Cooked	0.29 ± 0.00 aC	4.80 ± 0.15 aA	4.01 ± 0.03 aB	0.25 ± 0.01 bC
	**Sinapic acid**			
Raw	0.04 ± 0.00 A	0.02 ± 0.00 bB	0.16 ± 0.01 aC	Tr
Cooked	Tr	0.03 ± 0.00 aB	0.08 ± 0.00 bA	ND
	**Caffeic acid**			
Raw	Tr	3.04 ± 0.15 aB	3.62 ± 0.12 aA	0.02 ± 0.00 aC
Cooked	0.02 ± 0.00 C	3.16 ± 0.08 aB	3.46 ± 0.02 aA	0.00 ± 0.00 bC
	**p-coumaric acid**			
Raw	0.1 ± 0.00 bC	2.47 ± 0.08 aA	2.38 ± 0.06 aA	0.30 ± 0.01 aB
Cooked	0.29 ± 0.00 aC	2.65 ± 0.01 aA	2.37 ± 0.09 aB	0.27 ± 0.01 bC
	**3,4-Dihydroxybenzoic acid ^1^**			
Raw	0.97 ± 0.01 bC	1.21 ± 0.06 aB	1.76 ± 0.04 aA	0.95 ± 0.12 bC
Cooked	1.19 ± 0.01 aC	1.57 ± 0.03 aA	0.50 ± 0.00 bD	1.32 ± 0.03 aB
	**Total phenolic acids**			
Raw	1.51	11.07	11.90	1.66
Cooked	1.92	12.31	10.48	1.85
**Flavonoids**				
	**Catechin**			
Raw	4.96 ± 0.09 aC	5.00 ± 0.27 aC	7.96 ± 0.42 aB	9.58 ± 0.35 aA
Cooked	0.83 ± 0.03 bC	0.98 ± 0.02 bBC	1.48 ± 0.10 bA	1.07 ± 0.02 bB
	**Quercetin**			
Raw	2.39 ± 0.12 aC	9.04 ± 0.35 aB	17.75 ± 0.69 aA	2.39 ± 0.12 aC
Cooked	0.33 ± 0.03 bC	3.68 ± 0.17 bB	10.41 ± 0.48 bA	0.12 ± 0.02 bC
	**Luteolin**			
Raw	Tr	0.35 ± 0.01 aB	0.68 ± 0.01 aA	Tr
Cooked	Tr	0.07 ± 0.00 bB	0.14 ± 0.01 bA	Tr
	**Kaempferol**			
Raw	1.74 ± 0.01 aD	25.00 ± 0.43 aB	49.70 ± 0.47 aA	4.18 ± 0.12 aC
Cooked	1.06 ± 0.03 bC	14.39 ± 0.09 bB	23.42 ± 0.35 bA	0.36 ± 0.04 bD
	**Epicatechin**			
Raw	2.80 ± 0.11 aB	2.93 ± 0.07 aB	7.43 ± 0.51 aA	7.46 ± 0.56 aA
Cooked	0.89 ± 0.04 bB	0.52 ± 0.01 bC	1.60 ± 0.02 bA	0.81 ± 0.04 bB
	**Myricetin**			
Raw	0.05 ± 0.00 A	0.12 ± 0.00 aA	0.84 ± 0.55 aA	ND
Cooked	ND	0.07 ± 0.00 bB	0.21 ± 0.04 aA	ND
	**Taxifolin ^2^**			
Raw	3.19 ± 0.10 BC	4.88 ± 0.16 aB	5.93 ± 0.20 aA	3.55 ± 0.11 aC
Cooked	1.99 ± 0.01 bD	3.28 ± 0.04 bB	3.64 ± 0.06 bA	2.33 ± 0.01 bC
	**Total flavonoids**			
Raw	15.13	47.32	90.29	27.16
Cooked	5.10	22.99	40.90	4.69

**^1^** Gallic acid equivalents; **^2^** Quercetin equivalents; Tr: Trace; ND: Not Detected. Data are mean ± standard deviation (n = 9). Different lowercase letters in the columns represent significant differences in phenolic content between raw and cooked samples for each treatment. Different capital letters in the rows represent significant differences in phenolic content between treatments.

**Table 2:** Identification and concentration by UPLC-MS/MS (μg/g dry weight) of the insoluble phenolic fraction of common bean (*Phaseolus vulgaris* L.) of the Magnum variety, under different extraction treatments.

	Ultrasound	Viscozyme L^®^	Ultrasound + Viscozyme L^®^	Control
**Phenolic acids**				
	**Gallic acid**			
Raw	ND	0.05 ± 0.00 bB	0.08 ± 0.00 aA	ND
Cooked	0.17 ± 0.01 A	0.09 ± 0.00 aB	0.06 ± 0.00 bC	ND
	**Syringic acid**			
Raw	0.01 ± 0.00 aC	0.03 ± 0.00 bB	0.03 ± 0.00 bA	0.01 ± 0.00 aC
Cooked	0.01 ± 0.00 aC	0.04 ± 0.00 aB	0.05 ± 0.00 aA	0.01 ± 0.00 bC
	**Ferulic acid**			
Raw	0.62 ± 0.02 aC	4.17 ± 0.09 bA	3.7 ± 0.18 bB	0.76 ± 0.01 aC
Cooked	0.31 ± 0.00 bC	5.43 ± 0.09 aA	5.1 ± 0.07 aB	0.28 ± 0.00 bC
	**Sinapic acid**			
Raw	0.1 ± 0.00 B	0.03 ± 0.00 aC	0.12 ± 0.00 aA	Tr
Cooked	Tr	0.01 ± 0.00 bB	0.03 ± 0.00 bA	ND
	**Caffeic acid**			
Raw	0.05 ± 0.00 C	2.31 ± 0.08 aB	3.15 ± 0.06 aA	0.02 ± 0.00 C
Cooked	Tr	2.04 ± 0.06 aB	2.45 ± 0.02 bA	Tr
	**p-coumaric acid**			
Raw	0.43 ± 0.01 aB	2.36 ± 0.01 aA	2.41 ± 0.08 aA	0.50 ± 0.01 aB
Cooked	0.21 ± 0.00 bB	2.41 ± 0.13 aA	2.48 ± 0.18 aA	0.21 ± 0.03 bB
	**3,4-Dihydroxybenzoic acid ^1^**			
Raw	0.66 ± 0.05 aB	0.75 ± 0.03 bAB	0.95 ± 0.02 bA	0.49 ± 0.17 aB
Cooked	0.72 ± 0.01 aB	1.11 ± 0.02 aA	1.12 ± 0.01 aA	0.87 ± 0.10 aB
	**Total phenolic acids**			
Raw	1.87	9.70	10.44	1.78
Cooked	1.42	11.13	11.29	1.37
**Flavonoids**				
	**Catechin**			
Raw	7.67 ± 0.18 aB	7.88 ± 0.31 aB	10.23 ± 0.30 aA	8.22 ± 0.03 aB
Cooked	0.96 ± 0.03 bB	1.83 ± 0.08 bA	0.93 ± 0.06 bB	1.01 ± 0.09 bB
	**Quercetin**			
Raw	1.02 ± 0.05 BC	7.67 ± 0.13 aB	16.03 ± 0.01 aA	0.22 ± 0.03 aD
Cooked	0.49 ± 0.06 bC	2.53 ± 0.10 bB	5.01 ± 0.52 bA	0.07 ± 0.01 bC
	**Luteolin**			
Raw	Tr	0.67 ± 0.01 aB	0.91 ± 0.01 aA	Tr
Cooked	Tr	0.23 ± 0.01 bB	0.30 ± 0.01 bA	Tr
	**Kaempferol**			
Raw	5.70 ± 0.14 aC	37.35 ± 0.42 aB	59.65 ± 0.14 aA	3.89 ± 0.16 aD
Cooked	4.03 ± 0.08 bC	20.31 ± 0.07 bB	31.01 ± 0.16 bA	2.01 ± 0.14 bD
	**Epicatechin**			
Raw	8.38 ± 0.22 aB	4.56 ± 0.07 aD	9.80 ± 0.30 aA	6.44 ± 0.09 aC
Cooked	1.07 ± 0.03 bA	1.04 ± 0.01 bA	1.01 ± 0.03 bA	0.79 ± 0.12 bB
	**Myricetin**			
Raw	0.03 ± 0.00 aB	0.17 ± 0.01 aB	0.86 ± 0.10 aA	ND
Cooked	0.01 ± 0.00 bC	0.15 ± 0.00 aB	0.39 ± 0.06 bA	ND
	**Taxifolin ^2^**			
Raw	2.66 ± 0.09 aB	5.75 ± 0.11 aA	5.85 ± 0.18 aA	2.95 ± 0.08 aB
Cooked	1.88 ± 0.03 bC	4.28 ± 0.14 bA	3.93 ± 0.09 bB	1.76 ± 0.05 bC
	**Total flavonoids**			
Raw	25.46	64.05	103.33	21.72
Cooked	8.44	30.37	42.58	5.64

**^1^** Gallic acid equivalents; **^2^** Quercetin equivalents; Tr: Trace, ND: Not Detected. Data are mean ± standard deviation (n = 9). Different lowercase letters in the columns represent significant differences in phenolic content between raw and cooked samples for each treatment. Different capital letters in the rows represent significant differences in phenolic content between treatments.

**Table 3:** Effect of enzymatic treatment, ultrasound and the combination of ultrasound + enzymatic treatment on the antioxidant activity (μmol TE/g dry sample) of raw and cooked Peumo variety beans.

	Ultrasound	Viscozyme L^®^	US + Viscozyme L^®^	Control
**ABTS (μmol TE/g dry sample)**				
Raw	4.2 ± 0.36 aB	6.83 ± 0.55 aA	6.1 ± 0.65 aA	5.12 ± 0.6 aB
Cooked	2.01 ± 0.13 bC	2.55 ± 0.07 bA	2.41 ± 0.17 bAB	2.23 ± 0.17 bBC
**DPPH (μmol TE/g dry sample)**				
Raw	1.6 ± 0.09 aB	1.02 ± 0.14 aA	1.89 ± 0.10 aA	1.65 ± 0.15 aB
Cooked	0.96 ± 0.17 bA	0.94 ± 0.15 bA	0.99 ± 0.13 bA	0.10 ± 0.10 bA
**FRAP (μmol TE/g dry sample)**				
Raw	15.44 ± 1.23 aC	17.42 ± 0.45 aB	23.68 ± 1.19 aA	14.8 ± 0.74 aC
Cooked	5.98 ± 0.62 bC	12.49 ± 0.80 bA	8.06 ± 0.81 bB	7.15 ± 0.63 bBC
**ORAC (μmol TE/g dry sample)**				
Raw	0.89 ± 0.02 BC	1.22 ± 0.04 aA	1.16 ± 0.02 aA	1.02 ± 0.05 aB
Cooked	0.55 ± 0.10 bB	0.88 ± 0.05 bA	0.93 ± 0.03 bA	0.43 ± 0.04 bC

ABTS: 2,2′-azino-bis-(3-ethylbenzothiazolin-6-sulfonic acid); DPPH: 2,2-Diphenyl-1-Picrylhydrazyl; FRAP: Ferric Reducing Antioxidant Power Assay; ORAC: Oxygen Radical Absorbance Capacity; TE: Trolox equivalents; US: ultrasound. Data are the mean ± standard deviation (n = 9). Different lowercase letters in the columns represent significant differences between raw and cooked samples for each antioxidant activity method. Different capital letters in the rows represent significant differences between extraction types.

**Table 4:** Effect of enzymatic treatment, ultrasound and the combination of ultrasound + enzymatic treatment on the antioxidant activity (μmol TE/g dry sample) of raw and cooked Magnum variety beans.

	Ultrasound	Viscozyme L^®^	US + Viscozyme L^®^	Control
**ABTS (μmol TE/g dry sample)**				
Raw	3.6 ± 0.17 aB	5.69 ± 0.4 aA	5.86 ± 0.34 aA	3.31 ± 0.58 aB
Cooked	0.31 ± 0.18 bB	1.03 ± 0.23 bA	1.24 ± 0.22 bA	0.49 ± 0.07 bB
**DPPH (μmol TE/g dry sample)**				
Raw	1.53 ± 0.12 aB	2.31 ± 0.11 aA	2.27 ± 0.09 aA	1.65 ± 0.17 aB
Cooked	0.9 ± 0.08 bA	1.07 ± 0.17 bA	0.92 ± 0.08 bA	0.66 ± 0.05 bB
**FRAP (μmol TE/g dry sample)**				
Raw	13.19 ± 0.88 BC	24.12 ± 1.96 aA	24.05 ± 0.71 aA	15.65 ± 1.01 aB
Cooked	4.34 ± 0.68 bC	8.38 ± 0.61 bA	6.88 ± 0.66 bB	4.44 ± 0.31 bC
**ORAC (μmol TE/g dry sample)**				
Raw	1.08 ± 0.04 aB	1.22 ± 0.04 aA	1.23 ± 0.03 aA	1.02 ± 0.12 aB
Cooked	0.48 ± 0.04 bB	0.92 ± 0.04 bA	0.9 ± 0.07 bA	0.45 ± 0.01 bB

ABTS: 2,2′-azino-bis-(3-ethylbenzothiazolin-6-sulfonic acid); DPPH: 2,2-Diphenyl-1-Picrylhydrazyl; FRAP: Ferric Reducing Antioxidant Power Assay; ORAC: Oxygen Radical Absorbance Capacity; TE: Trolox equivalents; US: ultrasound. Data are the mean ± standard deviation (n = 9). Different lowercase letters in the columns represent significant differences between raw and cooked samples for each antioxidant activity method. Different capital letters in the rows represent significant differences between extraction types.

## Data Availability

The original contributions presented in this study are included in the article/Supplementary Materials. Further inquiries can be directed to the corresponding author.

## Supplementary Materials

The following supporting information can be downloaded at: https://www.mdpi.com/article/10.3390/plants15121823/s1, Table S1. Identification of phenolic compounds was carried out using multiple reaction monitoring (MRM) and authentic standards by UPLC-ESI-MS/MS.

## Abbreviations

The following abbreviations are used in this manuscript:

ABTS	2,2′-azino-bis-(3-ethylbenzothiazolin-6-sulfonic acid)
DPPH	2,2-Diphenyl-1-Picrylhydrazyl
FRAP	Ferric Reducing Antioxidant Power Assay
GAE	Gallic Acid Equivalents
ORAC	Oxygen Radical Absorbance Capacity
TE	Trolox Equivalents
TPC	Total Phenolic Content
UPLC-ESI-MS/MS	Ultra Performance Liquid Chromatography—Electrospray Ionization—Tandem Mass Spectrometry

## References

[1] Márquez K., Arriagada O., Pérez-Díaz R., Cabeza R.A., Plaza A., Arévalo B., Meisel L.A., Ojeda D., Silva H., Schwember A.R. (2024). Nutritional Characterization of Chilean Landraces of Common Bean. Plants.

[2] Moreno-Valdespino C.A., Luna-Vital D., Camacho-Ruiz R.M., Mojica L. (2020). Bioactive Proteins and Phytochemicals from Legumes: Mechanisms of Action Preventing Obesity and Type-2 Diabetes. Food Res. Int..

[3] Chen P.X., Tang Y., Marcone M.F., Pauls P.K., Zhang B., Liu R., Tsao R. (2015). Characterization of Free, Conjugated and Bound Phenolics and Lipophilic Antioxidants in Regular- and Non-Darkening Cranberry Beans (*Phaseolus vulgaris* L.). Food Chem..

[4] Hernández-Salazar M., Osorio-Díaz P., Loarca-Piña G., Reynoso-Camacho R., Tovar J., Bello-Pérez L.A. (2010). In Vitro Fermentability and Antioxidant Capacity of the Indigestible Fraction of Cooked Black Beans (*Phaseolus vulgaris* L.), Lentils (*Lens culinaris* L.) and Chickpeas (*Cicer arietinum* L.). J. Sci. Food Agric..

[5] Shahidi F., Hossain A. (2023). Importance of Insoluble-Bound Phenolics to the Antioxidant Potential Is Dictated by Source Material. Antioxidants.

[6] Durmuş M., Kelebek H., Selli S. (2024). Recovery, Bioactivity, and Utilization of Bioactive Phenolic Compounds in Citrus Peel. Food Sci. Nutr..

[7] Vilas-Franquesa A., Casertano M., Tresserra-Rimbau A., Vallverdú-Queralt A., Torres-León C. (2024). Recent Advances in Bio-Based Extraction Processes for the Recovery of Bound Phenolics from Agro-Industrial by-Products and Their Biological Activity. Crit. Rev. Food Sci. Nutr..

[8] Rosset M., Prudencio S.H., Beléia A.D.P. (2012). Viscozyme L Action on Soy Slurry Affects Carbohydrates and Antioxidant Properties of Silken Tofu. Food Sci. Technol. Int..

[9] Dueñas M., Hernández T., Estrella I. (2007). Changes in the Content of Bioactive Polyphenolic Compounds of Lentils by the Action of Exogenous Enzymes. Effect on Their Antioxidant Activity. Food Chem..

[10] Zhang J., Li M., Cheng J., Zhang X., Li K., Li B., Wang C., Liu X. (2021). Viscozyme L Hydrolysis and Lactobacillus Fermentation Increase the Phenolic Compound Content and Antioxidant Properties of Aqueous Solutions of Quinoa Pretreated by Steaming with α-Amylase. J. Food Sci..

[11] Januskevice V., Gomes A.M., Sousa S., Barbosa J.C., Vedor R., Martusevice P., Liaudanskas M., Zvikas V., Viskelis P., Cesoniene L. (2024). Phytochemical and Functional Diversity of Enzyme-Assisted Extracts from *Hippophae rhamnoides* L., *Aralia cordata* Thunb., and *Cannabis sativa* L. Antioxidants.

[12] González-Silva N., Nolasco-González Y., Aguilar-Hernández G., Sáyago-Ayerdi S.G., Villagrán Z., Acosta J.L., Montalvo-González E., Anaya-Esparza L.M. (2022). Ultrasound-Assisted Extraction of Phenolic Compounds from *Psidium cattleianum* Leaves: Optimization Using the Response Surface Methodology. Molecules.

[13] Chemat F., Rombaut N., Sicaire A.-G., Meullemiestre A., Fabiano-Tixier A.-S., Abert-Vian M. (2017). Ultrasound Assisted Extraction of Food and Natural Products. Mechanisms, Techniques, Combinations, Protocols and Applications. A Review. Ultrason. Sonochem..

[14] Falcão H.G., Handa C.L., Silva M.B.R., de Camargo A.C., Shahidi F., Kurozawa L.E., Ida E.I. (2018). Soybean Ultrasound Pre-Treatment Prior to Soaking Affects β-Glucosidase Activity, Isoflavone Profile and Soaking Time. Food Chem..

[15] Rojas-Vidal M.J., Varas Condori M.A., Arias-Santé M.F., Rhein S., Bridi R., Rincón-Cervera M.A., Nina N., Schmeda-Hirschmann G., Frías J., de Camargo A.C. (2026). Insoluble-Bound Phenolics of Selected Andean Common Bean Flours as Affected by Cooking: Characterization and Antioxidant Activity. Food Prod. Process. Nutr..

[16] Bautista-Expósito S., Vandenberg A., Dueñas M., Peñas E., Frias J., Martínez-Villaluenga C. (2022). Selection of Enzymatic Treatments for Upcycling Lentil Hulls into Ingredients Rich in Oligosaccharides and Free Phenolics. Molecules.

[17] Kim S.-M., Lim S.-T. (2016). Enhanced antioxidant activity of rice bran extract by carbohydrase treatment. J. Cereal Sci..

[18] Yusoff I.M., Mat Taher Z., Rahmat Z., Chua L.S. (2022). A Review of Ultrasound-Assisted Extraction for Plant Bioactive Compounds: Phenolics, Flavonoids, Thymols, Saponins and Proteins. Food Res. Int..

[19] Ling Y.Y., Sook Fun P., Yeop A., Yusoff M.M., Gimbun J. (2019). Assessment of Maceration, Ultrasonic and Microwave Assisted Extraction for Total Phenolic Content, Total Flavonoid Content and Kaempferol Yield from Cassia Alata via Microstructures Analysis. Mater. Today.

[20] Van Thanh H., Phi N.T.L., Khoi N.T., Hoan N.X., Van Hung P. (2023). Green Extraction and Biological Activity of Phenolic Extracts from Cashew Nut Testa Using a Combination of Enzyme and Ultrasound-Assisted Treatments. J. Sci. Food Agric..

[21] Van Hung P., Yen Nhi N.H., Ting L.Y., Lan Phi N.T. (2020). Chemical Composition and Biological Activities of Extracts from Pomelo Peel By-Products under Enzyme and Ultrasound-Assisted Extractions. J. Chem..

[22] López A., El-Naggar T., Dueñas M., Ortega T., Estrella I., Hernández T., Gómez-Serranillos M.P., Palomino O.M., Carretero M.E. (2013). Effect of Cooking and Germination on Phenolic Composition and Biological Properties of Dark Beans (*Phaseolus vulgaris* L.). Food Chem..

[23] Zhu L., Zhan C., Yu X., Hu X., Gao S., Zang Y., Yao D., Wang C., Xu J. (2024). Extractions, Contents, Antioxidant Activities and Compositions of Free and Bound Phenols from Kidney Bean Seeds Represented by “Yikeshu” Cultivar in Cold Region. Foods.

[24] Nina N., Theoduloz C., Paillán H., Jiménez-Aspee F., Márquez K., Schuster K., Becker L., Oellig C., Frank J., Schmeda-Hirschmann G. (2023). Chemical Profile and Bioactivity of Chilean Bean Landraces (*Phaseolus vulgaris* L.). J. Funct. Foods.

[25] de Camargo A.C., Concepción Alvarez A., Arias-Santé M.F., Oyarzún J.E., Andia M.E., Uribe S., Núñez Pizarro P., Bustos S.M., Schwember A.R., Shahidi F. (2022). Soluble Free, Esterified and Insoluble-Bound Phenolic Antioxidants from Chickpeas Prevent Cytotoxicity in Human Hepatoma HuH-7 Cells Induced by Peroxyl Radicals. Antioxidants.

[26] de Camargo A.C., Regitano-d’Arce M.A.B., Biasoto A.C.T., Shahidi F. (2016). Enzyme-Assisted Extraction of Phenolics from Winemaking by-Products: Antioxidant Potential and Inhibition of Alpha-Glucosidase and Lipase Activities. Food Chem..

[27] Ambigaipalan P., de Camargo A.C., Shahidi F. (2017). Identification of Phenolic Antioxidants and Bioactives of Pomegranate Seeds Following Juice Extraction Using HPLC-DAD-ESI-MSn. Food Chem..

[28] De Camargo A.C., de Souza Vieira T.M.F., Regitano-D’Arce M.A.B., Calori-Domingues M.A., Canniatti-Brazaca S.G. (2012). Gamma Radiation Effects on Peanut Skin Antioxidants. Int. J. Mol. Sci..

[29] Augusto T.R., Salinas E.S.S., Alencar S.M., D’arce M.A.B.R., de Camargo A.C., de Souza Vieira T.M.F. (2014). Phenolic compounds and antioxidant activity of hydroalcoholic extracts of wild and cultivated murtilla (Ugni molinae Turcz.). Food Sci. Technol..

