# Supplementary material for: Effects of Enzyme- and Ultrasound-Assisted Treatments on the Recovery of Insoluble-Bound Phenolic Antioxidants from Common Bean Flours
Source: Plants (Basel). 2026 Jun 12;15(12):1823. doi: 10.3390/plants15121823 (PMC13306405; doi:10.3390/plants15121823)
Supplement: Supplementary file 1 [file plants-15-01823-s001.zip › plants-4307792-supplementary.pdf]

Table S1. Identification of phenolic compounds was carried out using multiple reaction monitoring (MRM) and authentic standards by UPLC-ESI-MS/MS

MRM* Transition 1	DP	CE	CXP	MRM Transition 2	DP	CE	CXP
3,4-Dihydroxybenzoic acid (MW: 154.1, RT: 2.35)							
152.9** > 107.9***	−50	−26	−3	152.9** > 108.9***	−50	−16	−3
p-Coumaric acid (molecular weight: 164.2, RT: 5.36)							
162.9** > 119.0***	−70	−20	−5	162.9** > 119.0***	−70	−38	−25
Gallic acid (molecular weight: 170.12, RT: 1.20)							
168.9** > 124.9***	-70	-18	-7	168.9** > 78.9***	-70	-18	-7
Caffeic acid (Molecular weight: 180.2; RT: 4.23)							
178.9** > 135.0***	−70	−20	−5	178.9** > 133.9***	−70	−32	−7
Ferulic acid (Molecular weight: 194.2; RT: 5.87)							
193.0** > 134.0***	−55	−20	−7	193.0** > 177.9***	−55	−16	−15
Syringic acid (Molecular weight: 198.17; RT: 4.52)							
197.0** > 181.9***	-65	-18	-5	196.9** > 122.9***	-65	-30	-7
Sinapic acid (MW: 224.2, RT: 5.94)							
223.0** > 207.9***	−75	−18	−7	223.0** > 148.8***	−75	−26	−13
Kaempferol (MW: 286.24, RT: 9.43)							
284.9** > 184.9	-135	-36	-15	284.9** > 116.9	-135	-48	-3
Luteolin(MW: 286.24, RT: 7.97)							
285.0** > 133.0***	-125	-42	-5	285.0** > 150.9***	-125	-34	-11
Catechin (MW: 290.3, RT: 3.78)							
289.0** > 245.0***	-100	-22	-13	289.0** > 108.9***	-100	-30	-7
Epicatechin (MW: 290.3, RT: 3.77)							
289.0** > 244.9***	-110	-20	-19	289.0** > 109.0***	-110	-30	-7
Quercetin (MW: 302.2, RT: 8.27)							
301.0** > 150.9***	−15	−28	−13	301.0** > 178.8***	−15	−24	−11
Taxifolin (MW: 304.2, RT: 6.01)							
302.8 ** > 285.0***	-70	-14	-9	302.8 ** 125.0***	-70	-24	-13
Myricetin (MW: 318.24, RT: 4.9)							
316.9** > 150.9***	-105	-30	-7	316.9** > 178.9***	-105	-26	-9

* MRM, [multiple reaction monitoring]; ** Precursor ions m/z; ***Product ions m/z; CE, [collision energy]; CXP, [collision cell exit potential]; DP, [de-clustering potential]; RT, Retention time; MW: Molecular weight; RT, retention time in minutes.
